# Notes and Notices

**Published:** 1894-09

**Authors:** 


### NOTES AND NOTICES.

Dr. C. E. Fisher, President-elect of the Institute, has for many years been an active and aggressive man of the profession. Coming to the West at an early day, he located in Texas. There the opposition which he encountered in advancing Homœopathy only made him the stronger. After several years of work, editorial and medical, in Austin and San Antonio, he left the field and spent several years in the hospitals of Europe. Returning rich in experience, he established himself in Chicago, where he is building up a large practice, and has founded the *Medical Century*, a leading homœopathic journal. He also holds the chair of surgery in the Hering Medical College of Chicago. He is a clear thinker, a forcible speaker, and possesses very strong convictions. His manner in debate is impressive, and at the various medical meetings carries great weight. He is a successful parliamentarian, a good organizer, and, withal, full of tact. Twenty-one years ago he made application for membership in the American Institute of Homœopathy, and his application was tabled on account of his having matriculated from a young college, at that time not recognized by the Association. Subsequently he entered and graduated from other institutions and was admitted to membership. Last year he was, by unanimous vote, rehabilitated and given rank from the date of his original application. His standing is now within four years of being a senior. The office to which he has just been elected is the highest in the gift of the Homœopathic School of Physicians, and he will enter upon his duties on January 1st, 1895. Dr. Fisher is forty-one years old.

Dr. Frank Kraft, of Cleveland, Ohio, was unanimously elected Provisional Secretary. Dr. Kraft is very popular with the members of the Institute, and is at present editor of *The American Homœopathist*. He has been in the newspaper business—daily and medical—for a number of years, and is reporting the proceedings of the present Convention verbatim for various medical journals.

Glenmary Home, a private sanitarium for the homœopathic treatment of mental and nervous diseases, located at Owego, Tioga County, New York, has published a fine portrait of Hahnemann in the form of a cabinet photograph. A copy of this portrait adorns the office of this journal. We have been the recipients also of a fine framed picture of the home and surrounding landscape.

Dr. Jean Ida McKay has removed from Telluride, Colorado, to Lynchburg, Virginia. She has also entered the marriage relation, taking the surname Gliddon. We wish Dr. Gliddon much good fortune in her new position in life.

FOR SALE.—The Homœopathic Physician, from 1885 to 1892, complete; price, $10.00. *Medical Advance*, from 1887 to 1892, price, $10.00.

Also miscellaneous extra copies of odd numbers of both The Homœo-

PATHIC PHYSICIAN and *The Medical Advance.* Address Clarence M. Selfridge, M. D., 1322 Seventh Avenue, East Oakland, California.

THE ORGANON AND MATERIA MEDICA CLUB OF THE BAY CITIES OF CALIFORNIA is the title of a new homœopathic society started in San Francisco. The officers are as follows: President, J. M. Selfridge, M. D.; Vice-President, William Bœricke, M. D.; Secretary and Treasurer, W. E. Ledyard, M. D.; Censors, A. McNeil, M. D., O. Swayze, M. D., and C. M. Selfridge, M. D.

THE NUMERICAL STRENGTH OF THE DIFFERENT SCHOOLS OF MEDICINE in the United States is the title of a paper prepared by Dr. John K. Scudder, Secretary of the Eclectic Medical Institute, of Cincinnati, Ohio, and editor of *The Eclectic Medical Journal*, of Cincinnati, and published in the pages of that journal. It has been prepared with much care, time, and labor, and is probably more accurate than anything of the kind hitherto known.

According to this estimate, the total number of old-school physicians is 72,028; of Homœopathic physicians, 9,648; of Eclectics, 10,292; of Physico-medical, 1,553; of unclassified, 11,524.

MRS. ALBINA G. SMITH SANDERS, wife of Dr. J. C. Sanders, was buried from the family residence, No. 608 Prospect Street, Cleveland, Ohio, Monday afternoon, August 13th, 1894. The services were conducted by Rev. Dean Williams of Trinity cathedral. The burial service was at Lake View cemetery, where rest the remains of her father and mother and of her own children gone before.

By the death of Mrs. Sanders Cleveland society and her family have sustained a loss well-nigh inexpressible. As a wife, mother, and Christian woman she was beloved, esteemed, and admired. She was the mother of a large family, three of whom alone survive her, namely, Dr. J. Kent Sanders, Albina G. Sanders, and Franklyn P. Sanders. One who knew her intimately writes of her as follows: " Mrs. Sanders always seemed to me a most self-contained woman. She had rare dignity and self-control. Her manner was gracious and winning and the expression of her countenance was always most benignant. I always thought of her as the centre of attraction in her household, as the trusted counselor of her husband, the wise director of her children."—*Evening Post* of Cleveland, O.

THE F. A. DAVIS CO., the well-known publishing house of Philadelphia, will issue, in September, a work which will be most favorably received by the medical profession. It is entitled *Obstetric Surgery*, and is written by Drs. Egbert H. Grandin and George W. Jarman, gentlemen who, from their long connection with the largest and most widely known maternity hospital in the United States (The New York Maternity Hospital), are peculiarly fitted to expound the subject from the modern progressive standpoint of election.

There is no work in any language which deals with the surgical side of obstetrics so thoroughly as the present. The rules of obstetric asepis and antisepis are so described and simplified as to enable even the busy general practitioner to surround his patients with the same safeguards as are guaranteed in well-ordered hospitals. The subject of pelvimetry, without due regard to which modern obstetric surgery cannot exist, is most tersely and exhaustively treated of. The indications under which artificial abortion and the induction of premature labor properly fall are clearly exemplified. The limitations of the forceps and of version, and the beneficent results to be secured through timely resort to symphysiotomy and the Cæsarean section are stated with the accuracy which the marvelous progress of the past few years allows. The surgical aspects of the puerperal state are carefully described, and the concluding chapter deals with the surgical treatment of ectopic gestation.

The net price of the volume will be $2.50, and it will be printed in large, clear type, on excellent paper, and handsomely bound in extra cloth. The full-page plates, about fourteen in number, will be printed on fine plate paper in photogravure ink.

---

### FUN FOR DOCTORS.

Following the Ruling Custom.—"You say you don't know what's the matter with the man," said the young college graduate, "and I'm sure I don't. What'll we do?"

"Do?" said the fashionable physician, firmly; "why, we'll operate on him for appendicitis, of course."—*Chicago Record.*

A Truthful Advertiser.—A man advertises for a competent person to undertake the sale of a new medicine, and adds that it will prove highly lucrative to the undertaker.—*Bowersox's Budget.*

Passing the Time of Day.—"Hello, Quinine?"

"Why, Malaria, old boy, how are you? Shake."

Could Stand Anything.—Patient—"Aren't you making the medicine too strong for my stomach, doctor?"

Doctor—"Oh! no, I think not. By the way, what's this? *A Yellow Aster*. Have you been reading it?"

"Yes. I have just read it through a second time."

"Madam, your stomach will stand anything. Tablespoonful every half hour. Good morning!"—*Chicago Record.*

Needless Expense.—"You've been consulting a doctor, you say? What for?"

"I have a bad cold."

"A bad cold only, and gone to the expense of consulting a doctor? Why, you could have found nine out of every ten of your friends who would have given you a sure cure for it."—*New York Press.*

